# Forme Fruste Choledochal Cysts in Children: Clinical Presentation and Treatment Outcomes—A Retrospective Multicenter Study

**DOI:** 10.3390/children12060689

**Published:** 2025-05-28

**Authors:** Aleksandar Sretenović, Milan Slavković, Dragana Vujović, Polina Pavićević, Nenad Zdujić, Dražen Budimir, Zenon Pogorelić

**Affiliations:** 1 Department of Pediatric Surgery, University Children’s Hospital Belgrade, 11000 Belgrade, Serbia; 2 School of Medicine, University of Belgrade, 11000 Belgrade, Serbia; 3 Department of Paediatric Surgery, University Hospital of Split, 21000 Split, Croatia; 4 Department of Surgery, School of Medicine, University of Split, 21000 Split, Croatia

**Keywords:** forme fruste choledochal cyst, choledochal cyst, pancreaticobiliary malunion, fusiform choledochal cyst, type 1C choledochal cyst

## Abstract

**Purpose**: Forme fruste choledochal cyst (FFCC) is a choledochal cyst with minimal or no dilatation of the extrahepatic bile duct (EHBD) and is usually associated with an anomalous pancreaticobiliary junction (APBJ). While sharing similar symptoms, inflammation, and malignant potential with classic biliary cysts, FFCC is often overlooked on ultrasound. This paper aims to present the experience of two tertiary pediatric centers in managing FFCC. **Methods:** In this retrospective study, the clinical data of pediatric patients treated for FFCC at two tertiary pediatric surgical centers between 1 January 2008 and 31 December 2023 were analyzed. The primary outcome was the clinical success of the surgical procedure, defined by the resolution of symptoms and the absence of major complications. Secondary outcomes included postoperative complications, type and duration of surgical procedures, and length of hospital stay. All patients underwent biliary reconstruction via either Roux-en-Y hepatico-jejunostomy or hepatico-duodenostomy. Clinical outcomes, including postoperative complications and patient follow-up, were evaluated. **Results**: Fourteen children (9 girls, 5 boys; aged 18 months to 12 years) underwent surgical treatment of FFCC. The mean age at surgery was 5.3 ± 3.8 years, and the mean diameter of the common bile duct was 7.9 ± 1.2 mm. Thirteen patients underwent Roux-en-Y hepatico-jejunostomy, and one underwent hepatico-duodenostomy. Over a mean follow-up period of 6.2 ± 3.6 years, no cholangitis or anastomotic stricture cases were observed. Two patients (14.3%) experienced minor wound infections managed conservatively. **Conclusions:** FFCC remains a diagnostic challenge due to its subtle imaging findings and non-specific clinical presentation. However, once identified, surgical excision with biliary reconstruction, most commonly via Roux-en-Y hepatico-jejunostomy, is a safe and effective treatment with excellent long-term outcomes. Given the potential for serious complications if left untreated, FFCC should be actively considered in pediatric patients with unexplained pancreatitis or biliary symptoms, even in the absence of overt ductal dilatation.

## 1. Introduction

Biliary tract cysts are rare conditions for which the incidence ranges from 1 to 100,000–150,000 in the population of Western countries [1]. Todani’s classification of biliary tract cysts classifies them into five types, depending on location and size [2]. However, in 1985, Lilly et al. described a new entity that did not fit into the existing classification, which was then termed “forme fruste” choledochal cyst (FFCC) and subsequently added to the Todani classification under the designation Ic [2,3]. Forme fruste is a medical term that means an atypical or attenuated form of a disease or syndrome, with non-typical symptoms or characteristics present.

Patients with FFCC can have a clinical presentation of abdominal pain, recurrent fever, pancreatitis, and obstructive jaundice similar to a “classic” biliary cyst but without the characteristic cystic dilatation of the bile ducts. Namely, the dilatation of the common bile duct (CBD) in FFCC is discrete and so-called fusiform or spindle-shaped and is always associated with an anomalous pancreaticobiliary junction (APBJ) [3]. This anomalous junction between the pancreatic duct and the CBD occurs outside the duodenal wall and the sphincter of Oddi, rather than inside it, as in the normal configuration. Also, this anomalous junction is associated with functional spasm of the sphincter of Oddi. This configuration of the biliary-pancreatic junction enables continuous secretion from the pancreatic into the biliary duct, where the bile activates pancreatic enzymes and damages the biliary mucosa, which can lead to dilatation of the bile ducts [4]. Expressing the dilatation of bile ducts in children using dimensions is challenging due to the variability of duct diameter with age and the limited research on this topic. The maximum diameter of the normal extrahepatic biliary tract (EBT) in children ranges from 0.3 mm in premature infants to 4.4 mm in older children [5,6]. The diameter of the CBD in one study of the pediatric population (173 children aged 1 to 13 years) measured by ultrasound is 1.27 mm ± 0.67 mm [7]. Shimotakahara et al. defined an FFCC as one with a maximum diameter of less than 10 mm at the time of excision [8]. Based on these and other published values, the FFCC can be reasonably characterized by fusiform dilatation of the CBD within the range of approximately 2 mm to 10 mm, exceeding expected norms for age, yet lacking the overt cystic morphology of classic choledochal cysts. Another important parameter in describing these anomalies is the length of the common pancreatico-biliary duct, for which there is also a lack of data in the literature. According to Gulerud et al., the maximum normal length of the common canal in newborns and infants is 3 mm and increases to 5 mm in adolescents up to 15 years of age [4].

The clinical significance of FFCC is that patients with this condition exhibit similar symptoms, histologically confirmed inflammation, and malignant potential to patients with “classical” biliary tract cysts. Diagnosis is challenging if FFCC is not specifically considered, since cystic dilatation of the CBD or EBT may not be clearly visible on ultrasound.

In this paper, we present our experience with FFCC treatment and the outcomes observed in pediatric patients at two tertiary centers.

## 2. Patients and Methods

### 2.1. Patients

Patient data were collected as part of a retrospective study conducted from 1 January 2008 to 31 December 2023 at the Department of Pediatric Surgery, University Children’s Hospital, Belgrade, Serbia, and at the Department of Pediatric Surgery, University Hospital of Split, Croatia. The inclusion criteria encompassed pediatric patients (aged 0–17 years) who underwent surgery for FFCC and had at least one year of follow-up. Patients who had undergone surgery for other types of choledochal cysts, those over 17 years of age, or those with incomplete medical records were excluded. Ultimately, 14 patients met the inclusion criteria and were included in the analysis: 10 from the Department of Pediatric Surgery, University Children’s Hospital, Belgrade, and 4 from the Department of Paediatric Surgery, University Hospital of Split.

### 2.2. Outcomes of the Study

The primary outcome measure was the final result of the FFCC surgery. Secondary outcomes were the occurrence of early or late complications, the duration of surgery, the type of procedure, and the length of hospital stay.

### 2.3. Study Design

**Figure 1:**
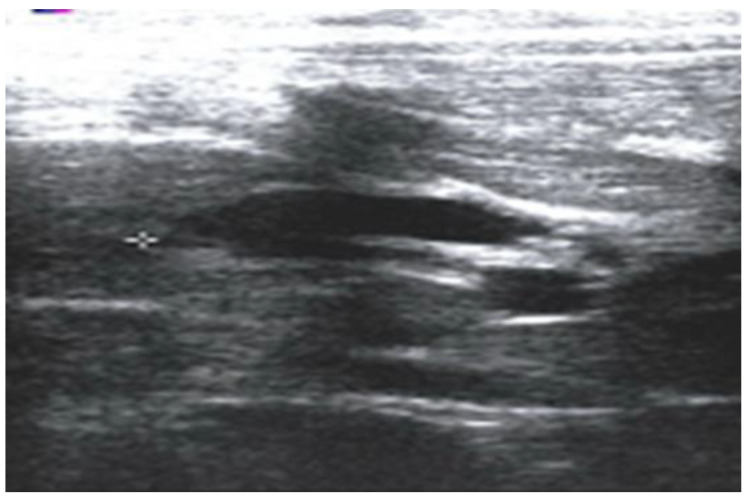
An abdominal ultrasound of a 2-year-old girl shows a dilated CBD with a diameter of 8.2 mm.

All patients enrolled in the study underwent either standard Roux-en-Y hepatico-jejunostomy or hepatico-duodenostomy. The FFCC is defined as a condition meeting all of the following criteria: (a) fusiform (non-cystic) dilatation of the CBD, with a diameter between 5 and 10 mm, exceeds the upper limit of normal for age but lacks classic cystic morphology; (b) an APBJ has been confirmed through magnetic resonance cholangiopancreatography (MRCP), endoscopic retrograde cholangiopancreatography (ERCP), or intraoperative cholangiography; (c) clinical presentation suggests biliary or pancreatic pathology (e.g., abdominal pain, recurrent fever, jaundice, and pancreatitis); (d) no other identifiable structural hepatobiliary anomalies. Standard diagnostic protocol included abdominal ultrasonography (Figure 1).

**Figure 2:**
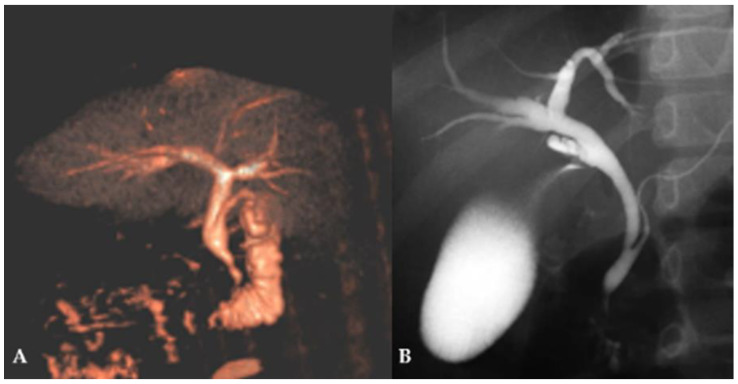
Diagnostic procedure in patient with suspected FFCC: (**A**)—MRCP in an 8-year-old girl, showing a dilated CBD with a diameter of 6.7 mm and an anomalous pancreaticobiliary junction; (**B**)—ERCP in a 12-year-old girl, showing a dilated CBD with a diameter of 8 mm and an anomalous pancreaticobiliary junction.

After confirmation of CBD dilatation, MRCP (Figure 2A) and, in selected cases, ERCP (Figure 2B) were performed depending on the clinical presentation.

**Figure 3:**
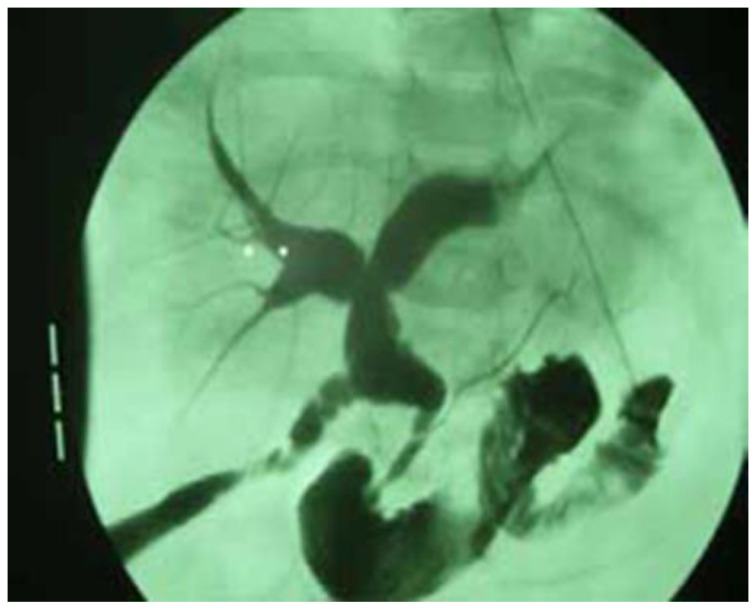
Intraoperative cholangiography in a 21-month-old boy, showing a dilated CBD with a diameter of 9 mm and an anomalous pancreaticobiliary junction.

Finally, prior to the surgical correction of the anomaly, an intraoperative cholangiography was performed in all patients to confirm the diagnosis (Figure 3).

The following parameters were recorded for each patient: age, gender, comorbidities, and laboratory markers: gamma-glutamyl transferase (GGT), aspartate aminotransferase (AST), alanine aminotransferase (ALT), alkaline phosphatase (AF), amylase, and lipase. In addition, the CBD diameter measured by MRCP, the early or late complications, the length of hospital stay, and the duration of follow-up were recorded for each patient.

### 2.4. Surgical Technique

All patients underwent either Roux-en-Y hepatico-jejunostomy or hepatico-duodenostomy. The patient is placed under general anesthesia. A sterile field is created, and prophylactic antibiotics are administered. The procedure begins with a midline laparotomy to expose the hepatoduodenal ligament. The dilated or abnormal segment of the bile duct is carefully dissected and excised, with preservation of the hepatic duct confluence. For hepatico-jejunostomy, a Roux loop of the jejunum (typically 40–50 cm distal to the ligament of Treitz) is brought up either retrocolically or antecolically and anastomosed to the hepatic duct in an end-to-side mucosa-to-mucosa hepatico-jejunostomy using absorbable sutures. A jejuno-jejunostomy is created 40–50 cm distal to the hepatico-jejunostomy to restore intestinal continuity. For hepatico-duodenostomy, the hepatic duct is directly anastomosed to the first or second part of the duodenum in an end-to-side fashion using absorbable sutures, ensuring a tension-free, mucosa-to-mucosa anastomosis. After ensuring hemostasis, a drain is placed near the anastomosis to monitor bile leakage, and the abdomen is closed in layers.

### 2.5. Postoperative Protocol and Follow-Up

After the surgical procedure, intravenous fluids were administered until oral intake was initiated. Oral intake typically began on the second or third postoperative day, depending on the surgeon’s decision, starting with liquids, followed by soft foods as tolerated. Drain removal is considered if there is no discharge or only minimal discharge, and no evidence of bile leakage. Paracetamol at a dose of 15 mg/kg or ibuprofen at a dose of 10 mg/kg was used for pain relief. In most cases, patients did not receive antibiotic therapy. Patients were discharged as soon as they tolerated oral intake, had stable vital signs, no signs of infection, good pain control on oral medication, and no signs of bile leakage or obstruction on imaging or laboratory tests. The children were followed up in our outpatient clinic 7 and 30 days after discharge and every 6 months thereafter to detect possible late complications.

### 2.6. Statistical Analysis

Statistical analysis was conducted using Python (Version 3.11; Python Software Foundation, Wilmington, DE, USA) with the SciPy library (Version 1.11.4; SciPy Developers, Tacoma, WA, USA) for Spearman correlation analysis. Data visualization was performed using Seaborn (Version 0.12.2; Beverly, MA, USA) and Matplotlib (Version 3.7.1; Matplotlib Development Team, Rutland, MA, USA). Descriptive statistics were employed in the study. Continuous variables were presented as means and standard deviations (SD), while categorical variables were reported as absolute numbers and percentages. A Spearman rank-order correlation was conducted to explore the potential relationship between the CBD diameter and symptom severity, using the number of presenting symptoms as a proxy for clinical severity, as no standardized scoring system for FFCC symptom burden exists. Additionally, the relationship between CBD diameter and total bilirubin level was analyzed. Correlation coefficients (ρ) and two-tailed *p*-values were reported, with a significance threshold set at *p* < 0.05.

## 3. Results

During the study period, a total of 14 pediatric patients who underwent surgery for FFCC were identified and included in the study cohort. In all 14 patients, APBJ was present, and the mean age at the time of surgery was 5.25 ± 3.82 years, with female predominance (*n* = 9, 64.3%). In the majority of the cases, the procedure of choice was Roux-en-Y hepatico-jejunostomy. Over a mean postoperative follow-up period of 6.2 years, two minor postoperative wound infections occurred (14.3%), both successfully managed with conservative treatment and classified as Clavien-Dindo Grade I complications. There have been no episodes of cholangitis or anastomotic stricture formation. The mean diameter of CBD at the time of surgery was 7.9 ± 1.16 mm (Table 1).

The most common presenting symptoms are shown in Table 2. The majority of the patients had elevated liver enzymes and mild to moderate increase of pancreatic enzymes. The summary of investigated laboratory parameters is shown in Table 3.

**Figure 4:**
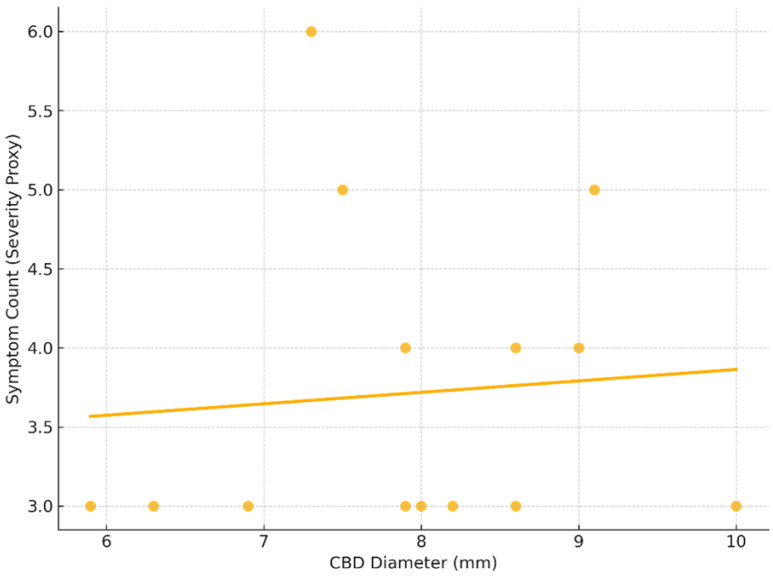
Correlation between CBD diameter and symptom severity. The Spearman correlation coefficient was ρ = 0.13, with a *p*-value = 0.66, indicating no statistically significant correlation.

A Spearman correlation analysis revealed a very weak positive correlation between CBD diameter and symptom count (ρ = 0.13), which was not statistically significant (*p* = 0.66) (Figure 4). This finding suggests that in pediatric FFCC cases, the degree of bile duct dilation does not reliably predict clinical symptom burden. These results underscore the diagnostic challenge of FFCC, as even patients with relatively mild ductal dilatation can present with multiple and severe symptoms.

**Figure 5:**
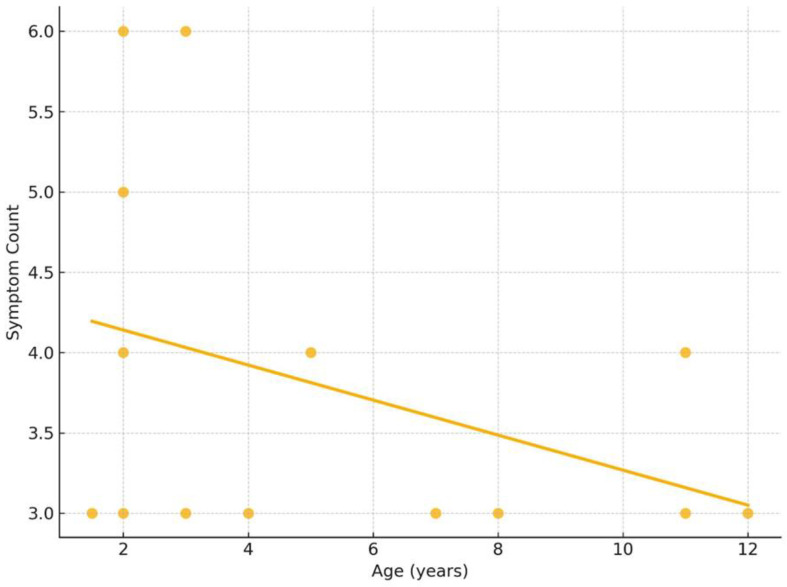
Correlation between age and symptom severity. The Spearman correlation coefficient was ρ = −0.30, with a *p*-value = 0.29, indicating no statistically significant correlation.

The Spearman correlation analysis between age and symptom count in pediatric FFCC cases revealed a weak negative correlation (ρ = −0.30), which was not statistically significant (*p* = 0.29) (Figure 5). This suggests no meaningful monotonic relationship between a child’s age and the severity (or number) of symptoms experienced.

**Figure 6:**
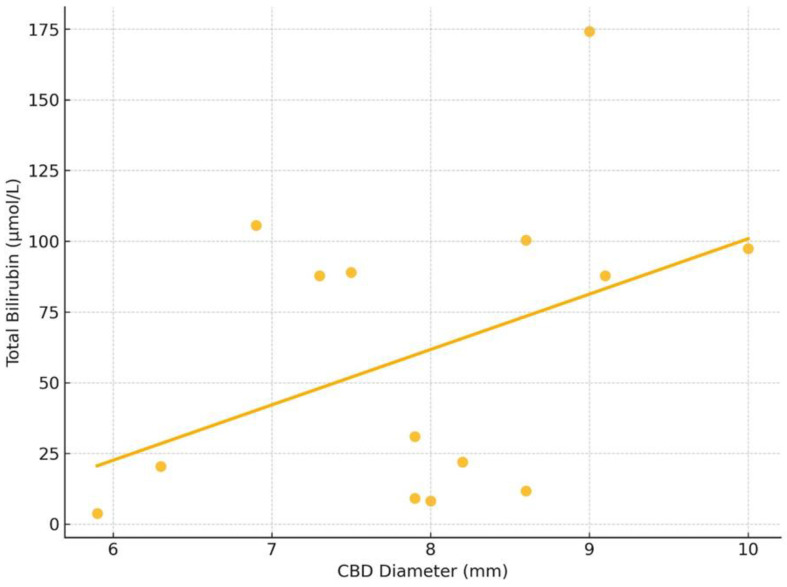
Correlation between CBD diameter and total bilirubin level. A Spearman correlation analysis revealed a moderate positive correlation between CBD diameter and total bilirubin level (ρ = 0.36), which was not statistically significant (*p* = 0.20).

The Spearman correlation between CBD diameter and total bilirubin level showed a moderate positive correlation (ρ = 0.36), but it was not statistically significant (*p* = 0.20) (Figure 6). This suggests a possible trend that larger duct diameter may be associated with higher bilirubin levels, though the result does not reach statistical significance.

## 4. Discussion

This study presents a retrospective review of 14 pediatric cases of FFCC treated surgically at two tertiary care centers over a 15-year period. All patients presented with non-specific gastrointestinal symptoms such as abdominal pain, nausea, vomiting, and recurrent fever—clinical features that often overlap with acute pancreatitis. Importantly, all cases showed radiologic and/or intraoperative evidence of an anomalous pancreaticobiliary junction (APBJ) and mild fusiform dilatation of the CBD, confirming the diagnosis of FFCC. In our series, hepatico-jejunostomy was the preferred method of reconstruction and was performed in the majority of cases. It led to excellent clinical results without long-term complications such as cholangitis or anastomotic stricture during an average follow-up period of 6.2 years. In the only case in which a hepatico-duodenostomy was performed, the choice of reconstruction was based on the intraoperative findings. The patient’s anatomy allowed a safe, tension-free anastomosis to the duodenum, and the operating surgeon chose this approach based on his clinical judgment. As this was a retrospective study, such decisions were made individually and were not guided by standardized selection criteria. Only two patients experienced minor wound infections, both of which were treated conservatively. Additionally, Spearman correlation analyses were performed to assess potential relationships between anatomical and clinical parameters. There was no statistically significant correlation between CBD diameter and symptom count or between age and symptom severity. A moderate but non-significant positive correlation was observed between CBD diameter and total bilirubin level, suggesting a possible trend that warrants further investigation. These results reinforce the diagnostic complexity of FFCC, where clinical presentation does not consistently align with measurable anatomical changes. Specifically, the favorable outcomes in our FFCC cohort, including low complication rates and lack of late biliary sequelae, are comparable to those reported for classic choledochal cysts with complete excision and bilioenteric reconstruction [8]. This reinforces our view that despite their subtle appearance, FFCCs should be treated with the same surgical rigor as more overt cystic variants.

Compared to the existing literature, our cohort has similar demographic characteristics. The mean age at surgery was 5.3 ± 3.8 years, which was exactly the same as the mean age of 5.9 years reported in studies by Shimotakahara et al. [8] and Fujishiro et al. [9]. Shimotakahara et al. described 15 pediatric patients with FFCC, pointed out the importance of early surgical intervention, and confirmed the long-term safety and efficacy of Roux-en-Y hepatico-jejunostomy at a median follow-up of 6.3 years [8]. The youngest patient in our series was 18 months old and thus among the earliest reported cases, comparable to the 14-month-old patient in the study by Barker et al., who emphasized the diagnostic ambiguity of FFCC due to its subtle clinical presentation and proposed the term “common biliopancreatic channel syndrome” [10]. In our cohort, females predominated (64.3%), which is consistent with the gender distribution reported by Wang et al. and Urushihara et al. who both found a female to male ratio of approximately 2:1 [11,12]. As for CBD diameter, our mean value of 7.9 ± 1.2 mm is slightly lower than that found in the pediatric FFCC cohort of Wang et al. (mean 8.6 mm) but similar to the range reported by Shimotakahara et al. (6–10 mm) [8,11]. This suggests a consistent anatomical threshold for FFCC diagnosis across studies, generally accepted as less than 10 mm. Aggarwal et al. emphasized the clinical heterogeneity of CBD measurements and recommended combining imaging techniques, such as 3D-MRCP, with clinical signs for the early detection of FFCC [13].

Surgical outcomes in our series were remarkably favorable, reflecting the low complication rates reported in other pediatric studies. Shimotakahara et al. observed no long-term complications in their study of 15 patients and emphasized that even in cases with minor ductal dilatation, complete excision and reconstruction are warranted to prevent malignant transformation [8]. Similarly, Urushihara et al. reported on 120 patients with various types of choledochal cysts, 49 of whom were classified as type Ic (including FFCC), most of whom underwent Roux-en-Y hepatico-jejunostomy. Long-term outcomes were excellent, and complication rates were low, especially when the anastomosis was performed at the confluence of the hepatic ducts [12]. Our rate of minor complications (14.3%) is lower than that reported by Wang et al., who observed postoperative complications in 27.3% of their 11 pediatric FFCC cases [11]. These results confirm that, when performed carefully, surgery for FFCC provides long-lasting symptom relief and minimizes the risk of late complications. Newer techniques such as robot-assisted excision have shown promising results, especially in large pediatric centers, although data are limited to small case series [14,15].

The diagnosis of FFCC is particularly challenging due to the subtle imaging features and the absence of classic cystic dilatation. While ultrasound remains the method of choice due to its accessibility, it often fails to detect an abnormal pancreaticobiliary junction or mild CBD dilatation, as demonstrated in the cohort of Wang et al. where ultrasound did not detect APBJ in any of the patients [11]. In contrast, MRCP provides non-invasive visualization of the biliary tract and is useful in children, but its sensitivity is limited as it only detects APBJ in 40.7% of cases in the same study. ERCP, although invasive and associated with risks such as pancreatitis and cholangitis, showed a sensitivity of 100% for the detection of APBJ and ductal abnormalities in pediatric FFCC patients [11]. For this reason, ERCP remains the most reliable tool for suspected FFCC and can serve a dual diagnostic and therapeutic purpose. Intraoperative cholangiography, which was used in all our cases, was crucial in confirming APBJ and ductal anatomy when preoperative imaging was inconclusive. As emphasized by Mishra et al., MSCT with 3D reconstruction can also help in the preoperative identification of vascular anomalies and support safe surgical planning [16]. Furthermore, evidence from clinical studies supports the routine use of intraoperative imaging, such as intraoperative cholangiopancreatography, to clarify the anatomy of the pancreaticobiliary junction and CBD in cases of diagnostic uncertainty during surgery for fusiform choledochal cysts, thereby reducing the risk of misdiagnosis [17]. An early MRCP with dynamic sequences is recommended for suspected pediatric biliary anomalies to improve the preoperative diagnostic yield in equivocal FFCC cases [18]. Despite these advances, there is no gold standard for the diagnosis of FFCC, and the diagnostic strategy should be individualized based on patient presentation, institutional resources, and surgical expertise.

Despite the good results obtained with open surgery, several recent studies have begun to evaluate the outcomes of laparoscopic versus open excision for type I choledochal cysts, with comparable efficacy and a trend toward shorter recovery times, although long-term data are still sparse [19,20]. An additional aspect of FFCC is the evolving understanding of pathogenesis and the implications for long-term risk stratification. Kowalski et al. in their study emphasized the importance of complete surgical resection in pediatric FFCC to prevent recurrent pancreatitis and long-term biliary complications, confirming our findings [21]. Hulscher et al. conducted an international Delphi survey among pediatric hepatobiliary specialists, highlighting the growing recognition of congenital biliary malformations in children and emphasizing the limitations of existing classification systems. The study advocates for more nuanced diagnostic frameworks that account for subtle anatomical variations, such as fusiform FFCC, to improve clinical management and surgical decision-making [22]. MRCP with 3D reconstruction has shown superior diagnostic yield compared to conventional imaging in identifying subtle biliary malformations in children, including fusiform choledochal cysts. These findings underscore the value of incorporating advanced imaging modalities into routine diagnostic pathways for congenital hepatobiliary disorders [23]. From a mechanistic perspective, Sugiyama and Atomi showed that an abnormal pancreaticobiliary junction can cause chronic damage to the biliary epithelium by enzyme reflux even in the absence of macroscopic cyst formation, supporting the rationale for surgical correction even in mild cases [24]. Matsumoto et al. also demonstrated abnormal motility of the sphincter of Oddi in patients with APBJ, contributing to the pathological reflux that characterizes the FFCC process and highlighting the functional as well as anatomical basis for surgery [25].

While choledochal cysts in adults have traditionally been associated with malignant transformation, recent evidence suggests that pediatric patients are not exempt from this risk, especially in the presence of APBJ and chronic biliary obstruction. Continued reflux of pancreatic enzymes into the bile duct has been shown to cause mucosal damage even in very young patients, leading to progressive ductal dilatation and persistent inflammation, the key factors implicated in carcinogenesis. Recent case reports and pathological studies support this concern. For example, a case of cholangiocarcinoma from a perforated choledochal cyst in a three-year-old boy was recently described [26]. In addition, in a retrospective study from Asan Medical Center, biliary intraepithelial neoplasia (BilIN)—a recognized precancerous condition—was found in 4.5% of samples from pediatric choledochal cysts, including some without obvious cystic dilatation [27]. Moreover, chronic inflammation due to uncorrected reflux of pancreatic enzymes has been mechanistically linked to malignant transformation of the biliary epithelium, as shown in recent molecular and clinical studies [28]. These findings highlight the malignant potential of choledochal abnormalities in childhood and emphasize the critical importance of early diagnosis and definitive surgical treatment. In particular, fibrous or focal fusiform choledochal cysts, which do not have distinct cystic features, have a similar pathophysiologic basis to APBJ and are, therefore, considered to have a comparable oncogenic risk. Early excision with bilioenteric reconstruction is essential not only for the relief of symptoms but also as a preventive strategy against future malignancy.

This study underscores the need for heightened clinical vigilance among pediatricians and pediatric surgeons when evaluating children with recurrent pancreatitis, unexplained abdominal pain, or intermittent jaundice, even in the absence of overt biliary dilation on ultrasound. FFCC should be actively considered in such cases, and further evaluation with MRCP or ERCP should be pursued when laboratory or clinical findings suggest biliary or pancreatic involvement. Given the demonstrated safety and long-term effectiveness of surgical intervention, early referral to specialized centers for diagnostic workup and surgical management is recommended. Adopting this approach may prevent chronic complications and reduce the risk of malignant transformation associated with delayed diagnosis.

Despite its strengths, our study has some limitations. The retrospective design and small sample size limit the generalizability of our results and preclude statistical comparison with larger datasets. Diagnostic methods were not standardized in all cases; some patients underwent both MRCP and ERCP, while others underwent only ultrasonography followed by intraoperative cholangiography. In addition, although the follow-up time was generally adequate, some patients had a shorter postoperative observation time, which may have underestimated delayed complications. The single case of hepatoduodenostomy also limits our ability to evaluate alternative surgical approaches, such as laparoscopic or robotic techniques, which are gaining increasing attention in the recent pediatric surgical literature [14,15].

These findings underscore the importance of maintaining a high index of suspicion for FFCC in pediatric patients with recurrent or unexplained biliary and pancreatic symptoms. When initial imaging is inconclusive, the timely use of advanced modalities and referral to specialized centers can enhance diagnostic accuracy and facilitate optimal surgical planning. The diagnostic criteria, surgical approach, and outcome data presented in this study may serve as a reference point for the development of standardized definitions and protocols in future prospective multicenter studies aimed at optimizing the diagnosis and treatment of FFCC in children.

## 5. Conclusions

In conclusion, early detection and prompt surgical management of FFCC with Roux-en-Y hepatico-jejunostomy, guided by accurate imaging and experienced surgical technique, result in excellent long-term outcomes and minimize the risk of complications and malignant transformation in pediatric patients.

## Figures and Tables

**Table 1:** Summary of clinical characteristics, diagnostics, surgical management, and follow-up in pediatric FFCC patients (*n* = 14).

Parameter	Value/Description
Number of patients	14
Gender	9 Female (64.3%), 5 Male (35.7%)
Mean age at surgery	5.25 ± 3.82 years (range: 1.5–12)
Surgical procedure	13 Roux-en-Y hepatico-jejunostomy (92.9%), 1 hepatico-duodenostomy (7.1%)
Mean CBD diameter	7.9 ± 1.16 mm (range: 5.6–10 mm)
Diagnostic modalities used	US: All patients; MRCP: Most cases; ERCP: Selected cases; Intraoperative cholangiography: All patients
Complications	2 wound infections (14.3%), managed conservatively
Cholangitis/Stricture	0 cases
Mean follow-up duration	6.2 ± 3.6 years (range: 8 months to 14 years)
Long-term outcomes	No late complications, no recurrence of symptoms

Abbreviations: FFCC—Forme Fruste Choledochal Cyst; CBD—Common Bile Duct; US—Abdominal ultrasound; MRCP—Magnetic Resonance Cholangiopancreatography; ERCP—Endoscopic Retrograde Cholangiopancreatography.

**Table 2:** Frequency of presenting symptoms in pediatric FFCC patients (*n* = 14).

Symptom	Number of Patients (n)	Percentage (%)
Abdominal pain	11	78.6
Nausea	9	64.3
Fever (mild or recurrent)	8	57.1
Vomiting	7	50.0
Jaundice	7	50.0
Failure to thrive	4	28.6
Pancreatitis	2	14.3
Diarrhoea	1	7.1
Fussiness	1	7.1
Sleeplessness	1	7.1
Refusal to eat	1	7.1

**Table 3:** Mean laboratory values at admission in pediatric FFCC patients (*n* = 14).

Laboratory Parameter	Mean ± SD	Unit
Aspartate Transaminase (AST)	162.1 ± 112.8	U/L
Alanine Transaminase (ALT)	222.6 ± 146.5	U/L
Gamma-glutamyl Transferase (GGT)	234.7 ± 129.3	U/L
Alkaline Phosphatase (AF)	333.5 ± 163.7	U/L
Total Bilirubin	63.4 ± 53.2	µmol/L
Direct Bilirubin	38.1 ± 39.2	µmol/L
Amylase	1103.1 ± 1248.6	U/L
Lipase	2323.6 ± 2973.5	U/L

Abbreviations: SD—Standard Deviation.

## Data Availability

The raw data supporting the conclusions of this article will be made available by the authors on request due to privacy and ethical reasons.
